# Preservation, Characterization and Exploitation of Microbial Biodiversity of Agri-Food and Environmental Interest

**DOI:** 10.3390/microorganisms8121938

**Published:** 2020-12-07

**Authors:** Ilaria Mannazzu, Marilena Budroni, Giacomo Zara, Severino Zara

**Affiliations:** Department of Agricultural Sciences, University of Sassari, Viale Italia 39A, 07100 Sassari, Italy

This Special Issue collects original contributions in the form of review or research articles, dealing with different aspects of the preservation, characterization and exploitation of the biodiversity of bacteria, yeast, algae and filamentous fungi of different origins.

The preservation of microbial biodiversity, besides being essential for the maintenance of life on Earth, is the first step towards the implementation of biotechnological processes aimed at the production of valuable goods and services of public utility. Accordingly, De Vero et al. [1] reported on the fundamental role of culture collections and microbial biological resource centers in the preservation of microbial biodiversity. Interestingly, microbial repositories play a role not only in the conservation of microbial species, genes, enzymes and metabolites with present and future biotechnological application, and in their management and circulation, but also in the preservation of local heritages. In this context, Feghaly et al. [2] described the isolation and characterization of the *Saccharomyces cerevisiae* population of Merwah, an ancient native grape variety of Lebanon, one of the eldest wine regions in the word. By preserving these yeasts and selecting possible starters for Merwah valorization, these authors contributed to the preservation of a traditional wine with cultural and economic value.

Besides being preserved, microbial diversity needs to be characterized in order to elucidate the composition of the microbial communities associated with the substrates of interest and to recognize their ecological role in these substrates. Thus, some of the studies of this Special Issue deal with the characterization of microbial diversity associated to food substrates, either to gather information on its richness or to evaluate its changes in response to environmental factors. In particular, Fancello et al. [3] explored the biodiversity of olive oil, a harsh environment for microbial growth. These authors reported that the oil microflora is poor from a numerical point of view, but rich in biodiversity and includes a number of bacterial species with a wide range of possible future applications that comprehend the industrial bioconversion of lipids, fats, and oils into high-value products, the detoxification of by-products of the agro-industry and the utilization as plant growth-promoting bacteria and substitutes of chemical fertilizer. Agarbati et al. [4] evaluated the impact of different fungicide treatments (organic vs. conventional) on the quali-quantitative composition of the yeast community of grape berries. In particular, these authors reported that, in respect to organic, conventional treatments result in a general loss of yeast biodiversity with the enrichment of oxidative yeast species. Considering that the grapes microbiota impacts on the final composition of wine, they concluded that the vineyard farming system influences wine fermentation.

The characterization of food microflora is aimed also at elucidating the composition of the microbial communities involved in the spoilage of fresh food. In this context, Wang et al. [5] observed that the storage temperature impacts on the bacterial communities residing on the fruit bodies of *Volvariella volvacea*, the most common edible fungus in the world. In particular, these authors observed that it is possible to counteract the growth of decay-causing bacteria by lowering storage temperature at 15 °C. Leneveu-Jenvrin et al. [6] characterized the diversity of yeast and molds that proliferate on pineapple in order to individuate the microbial species that trigger the decay of this fruit.

The exploitation of microbial diversity is generally aimed at developing sustainable biotechnological processes. In this Special Issue, the works regarding the application of microbial biodiversity are aimed at different purposes spanning from the development of circular economy, through the valorization of by-products or of significant products in rural economy, to research purposes. In particular, Da Silva et al. [7] reviewed the different strategies utilized for the cultivation and processing of heterotrophic microalgae for the valorization of low-cost substrates through the production of lipid ω-3-rich compounds and reported on the utilization of all microalgal fractions to increase the economic sustainability of the process. Szotkowski et al. [8] described the utilization of carotenogenic yeasts for the valorization of animal waste fat through the production of high value multifunctional biomasses. Dimitrellou et al. [9] analyzed the behavior of commercial and probiotic starter strains of lactic acid bacteria to promote the industrial exploitation of goat milk through the production of new functional dairy products. Finally, Balaban et al. [10] applied an evolutionary engineering approach to increase the genetic diversity of *S. cerevisiae* and obtain a population of iron resistant mutants. In this work, the exploitation of this artificially induced biodiversity was finalized to highlight a mutant on which to study the cellular mechanisms involved in iron homeostasis.

In conclusion, we believe that this Special Issue, which gathers insights into the preservation, characterization and exploitation of the biodiversity of microorganisms associated to different substrates, will be of interest both for the scientific community and the productive world.

## References

[1] De Vero L., Boniotti M.B., Budroni M., Buzzini P., Cassanelli S., Comunian R., Gullo M., Logrieco A.F., Mannazzu I., Musumeci R. (2019). Preservation, Characterization and Exploitation of Microbial Biodiversity: The Perspective of the Italian Network of Culture Collections. Microorganisms.

[2] Feghali N., Albertin W., Tabet E., Rizk Z., Bianco A., Zara G., Masneuf-Pomarede I., Budroni M. (2019). Genetic and Phenotypic Characterisation of a *Saccharomyces cerevisiae* Population of ‘Merwah’ White Wine. Microorganisms.

[3] Fancello F., Multineddu C., Santona M., Deiana P., Zara G., Mannazzu I., Budroni M., Dettori S., Zara S. (2019). Bacterial Biodiversity of Extra Virgin Olive Oils and Their Potential Biotechnological Exploitation. Microorganisms.

[4] Agarbati A., Canonico L., Mancabelli L., Milani C., Ventura M., Ciani M., Comitini F. (2019). The Influence of Fungicide Treatments on Mycobiota of Grapes and Its Evolution During Fermentation Evaluated by Metagenomic and Culture-Dependent Methods. Microorganisms.

[5] Wang X., Liu S., Chen M., Yu C., Zhao Y., Yang H., Zha L., Li Z. (2019). Low Temperature (15 °C) Reduces Bacterial Diversity and Prolongs the Preservation Time of *Volvariella volvacea*. Microorganisms.

[6] Leneveu-Jenvrin C., Quentin B., Assemat S., Horau M., Meile J.-C., Remize F. (2020). Changes of Quality of Minimally-Processed Pineapple (*Ananas comosus*, var. ‘Queen Victoria’) during Cold Storage: Fungi in the Leading Role. Microorganisms.

[7] Lopes Da Silva T., Moniz P., Silva C., Reis A. (2019). The Dark Side of Microalgae Biotechnology: A Heterotrophic Biorefinery Platform Directed to ω-3 Rich Lipid Production. Microorganisms.

[8] Szotkowski M., Byrtusova D., Haronikova A., Vysoka M., Rapta M., Shapaval V., Marova I. (2019). Study of Metabolic Adaptation of Red Yeasts to Waste Animal Fat Substrate. Microorganisms.

[9] Dimitrellou D., Salamoura C., Kontogianni A., Katsipi D., Kandylis P., Zakynthinos G., Varzakas T. (2019). Effect of Milk Type on the Microbiological, Physicochemical and Sensory Characteristics of Probiotic Fermented Milk. Microorganisms.

[10] Balabam B.G., Yilmaz U., Alkim C., Topaloglu A., Kisakesen H.I., Holyavkin C., Cakar Z.P. (2019). Evolutionary Engineering of an Iron-Resistant *Saccharomyces cerevisiae* Mutant and Its Physiological and Molecular Characterization. Microorganisms.

